# Chemical Heating for Minimally Instrumented Point-of-Care (POC) Molecular Diagnostics

**DOI:** 10.3390/bios14110554

**Published:** 2024-11-13

**Authors:** Michael G. Mauk, Felix Ansah, Mohamed El-Tholoth

**Affiliations:** 1 Department of Mechanical Engineering and Applied Mechanics, University of Pennsylvania, Philadelphia, PA 19104, USA; mmauk@seas.upenn.edu; 2 West African Centre for Cell Biology of Infectious Pathogens (WACCBIP), University of Ghana, Legon, Accra P.O. Box LG 54, Ghana; felansah@ug.edu.gh; 3 Department of Virology, Faculty of Veterinary Medicine, Mansoura University, Mansoura 35516, Egypt; 4 Health Sciences Division, Higher Colleges of Technology, Al Ain Zakhir Campus, Abu Dhabi 17155, United Arab Emirates

**Keywords:** chemical heating, self-heating, POC (point-of-care), diagnostics, isothermal nucleic acid amplification test (NAAT)

## Abstract

The minimal instrumentation of portable medical diagnostic devices for point-of-care applications is facilitated by using chemical heating in place of temperature-regulated electrical heaters. The main applications are for isothermal nucleic acid amplification tests (NAATs) and other enzymatic assays that require elevated, controlled temperatures. In the most common implementation, heat is generated by the exothermic reaction of a metal (e.g., magnesium, calcium, or lithium) with water or air, buffered by a phase-change material that maintains a near-constant temperature to heat the assay reactions. The ability to incubate NAATs electricity-free and to further to detect amplification with minimal instrumentation opens the door for fully disposable, inexpensive molecular diagnostic devices that can be used for pathogen detection as needed in resource-limited areas and during natural disasters, wars, and civil disturbances when access to electricity may be interrupted. Several design approaches are reviewed, including more elaborate schemes for multiple stages of incubation at different temperatures.

## 1. Introduction

This review surveys various approaches and implementations of chemical heating for minimally instrumented (electricity-free) POC medical diagnostic devices. Generally, these self-heating devices utilize a self-contained exothermic chemical reaction initiated at the time of use, along with a phase-change material, to produce controlled heating as needed in various steps of diagnostic assays, and particularly for molecular diagnostics, i.e., enzymatic nucleic acid amplification tests (NAATs).

**Figure 1:**
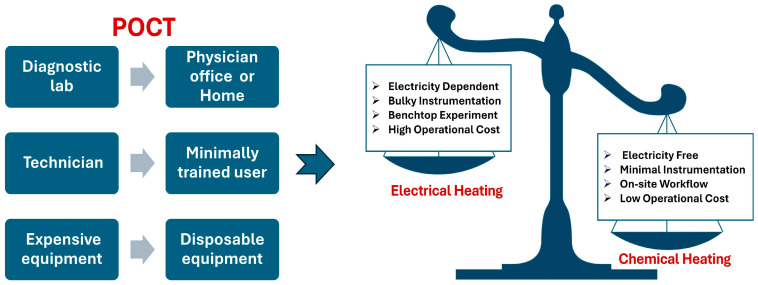
Electrical heating vs. chemical heating for point-of-care testing (POCT).

For comparison and as an ideal for other POC tests, lateral flow immunoassay strips, such as home pregnancy tests and similar rapid tests for infectious diseases, serve as the foremost example of pervasive, inexpensive, single-use (disposable), electricity-free diagnostic devices. These products are sold over the counter and can be used without any training or technical knowledge, are completely autonomous, and require no supporting instrument or equipment. In contrast, many other—if not most—POC diagnostic devices, especially those based on NAATs—require some form of temperature-regulated heating [1]. This implies a source of electricity, such as a battery or plug-in power supply, as electrical heating is the preferred means of temperature control. Since nucleic acid-based tests are generally more sensitive and specific than immunoassays [2], there is an incentive to make NAATs more like lateral flow strips with regard to simplicity, autonomous operation, and minimal instrumentation (Figure 1). One approach to this end is to use a chemical reaction to produce the required heat. This is no doubt inspired by so-called “meals ready-to-eat” (MRE) used by soldiers and outdoorsmen, wherein a packet of ‘fuel’ in powder form is mixed with water to bring food or beverages to near-boiling point temperatures [3], although there is not much temperature regulation other than being limited by the temperature of the boiling point of water. Another related consumer technology is hand warmers [4,5], which comprise a palm-sized pouch of sodium acetate that is melted by immersion in hot water and thereafter maintains a constant temperature (~50 °C) as the sodium acetate slowly (~30 min) recrystallizes.

Chemical heating for diagnostics is promoted by the advent of isothermal NAATs, such as Loop-mediated Amplification (LAMP) and Recombinase Polymerase Amplification (RPA)—reviewed recently in [6]. Long-established laboratory methods based on PCR (polymerase chain reaction) need benchtop instruments that provide precise (±0.5 °C) thermal cycling, peak cycle temperatures over 90 °C, and rapid heating and cooling (~±10 °C/s). In contrast, LAMP and RPA assays require merely constant-temperature incubation at approximately 65 °C (for LAMP) or 37–42 °C (for RPA) and thus are well suited for chemical heating [7,8,9].

The interest in electricity-free heating in POC tests stems from several considerations. There are an estimated 770 million people in the world without electricity, mostly in Sub-Saharan Africa [10], and in many areas of the world electric supply is unreliable with frequent outages. These resource-limited areas are where POC diagnostics are most needed due to the lack of medical infrastructure. Moreover, during natural disasters, civil disturbances, and wars, access to electricity may be interrupted. Chemical heating with phase-change materials also eliminates the need for a heater element, temperature sensor, and control electronics, considerably simplifying the device. Many proposed POC diagnostic systems utilize smartphone platforms for optical detection (cellphone cameras), computation, data logging, communication, GPS, and operator interfaces. However, electrical heating imposes an excessive demand on smartphone batteries, limiting the number of tests. Thus, smartphone-based diagnostics can also benefit from incorporating chemical heating to conserve battery power and reduce or eliminate peripheral instrumentation, such as electric heaters and temperature sensors [11,12].

The operational basis of chemical heating is to initiate an exothermic reaction with preloaded stored reagents (the ‘fuel’), typically in powder or solid form, by adding water or another solvent or exposing it to air. The reaction melts a phase-change material (PCM) that is an important medium for temperature control. The PCM dissipates its latent heat as it resolidifies and maintains a constant temperature, i.e., its melting point, for some prolonged time (~20 to 60 min) until it completely solidifies [13,14]. A sample in thermal contact with the PCM is thus incubated at the PCM melting point. Since a wide range of melting points are available with various phase-change materials, the incubation temperature can be adjusted accordingly. For LAMP, there is an incubation temperature window ranging from approximately 60 to 65 °C; however, the optimum amplification efficiency of LAMP may be sensitive within ±1 °C. RPA operates best in the range of 37 to 42 °C [8,15,16,17].

## 2. Chemical Heating Reactions

The main advantages of isothermal amplification assays over traditional molecular assays (such as PCR) are their constant incubation temperature, high amplification efficiency, and tolerance to contaminants and inhibitors. These factors reduce the complexity, cost, and power consumption of equipment, allowing diagnosis with minimal instrumentation [18]. The primary isothermal amplification assays used for point-of-care detection with chemical heating include the Recombinase Polymerase Assay (RPA) at approximately 37 °C, Helicase-Dependent Amplification (HAD) at around 65 °C, and Loop-Mediated Isothermal Amplification (LAMP) at about 65 °C [19].

In one of the earliest reports of chemical heating for an NAAT, LaBarre et al. [20,21] employed a CaO (77 g) + H_2_O (18 g) reaction, along with a proprietary PCM (65 °C melting point), to heat a LAMP assay at 62 to 65 °C for 45 min. Three 200-µL sample tubes (with 50 µL of LAMP reaction) were embedded in ~14 cm^3^ of PMC, which was melted by the surrounding chemical heating reaction. They used a modified thermal bottle as a container. The heat transfer from the PCM to the reaction tubes was enhanced by a honeycomb aluminum structure. Singleton et al. [22] discussed the use of other exothermic reactants, including MgFe + H_2_O, iron + air, sodium acetate, and other PCMs with melting points in the 58 to 68 °C range from various manufacturers. An electricity-free point-of-care molecular diagnostic system for HIV-1 using RT-LAMP (reverse transcription LAMP) in PCR tubes incubated at 62 °C and heated with water added to MgFe, all of which were contained in a thermos bottle, has also been described [23,24,25].

A colorimetric tube-based LAMP test using Mg:Fe chemical heating was reported [26]. Buser et al. [27] described precision chemical heating using a smaller-volume system (insulating blocks in place of a thermos bottle) and a wick to control the addition of water to the exothermic reagents.

One of the most commonly used chemical heating reactions is based on Mg:Fe (1%) powder with a small amount of NaCl, which is initiated by the addition of water. The exothermic reaction is

$$\mathrm{Mg}(\mathrm{Fe})(s)+2\;H_{2}O\;(𝓁)\;\text{→}\;\mathrm{Mg}(\mathrm{OH}{)}_{2}\;(s)+H_{2}\;(g)\;\;\;\;\;\;\;\Delta H≅-14600\frac{Joules}{g-Mg}$$

The Fe component of the alloy reacts in a secondary galvanic corrosion reaction (with NaCl), preventing MgO formation that would otherwise inhibit the reaction [26,28]. (One caveat is that many online sources of MgFe pouches are military surplus and may well have been warehoused for more than ten years. Evidently, some premature oxidation may occur, perhaps due to packaging being permeable to air and moisture, and the fuel may lose much of its potency. Consistency of results can be obtained by first testing samples from suppliers and pooling the materials in large, well-mixed batches.)

Other reactions used for chemical heating include adding water to calcium oxide,

$$\mathrm{CaO}(s)+H_{2}O\;(l)\to {\mathrm{Ca(OH)}}_{2}\;(s)\text{,}$$

adding water to calcium chloride,

$${\mathrm{CaCl}}_{2}(s)+2\;H_{2}O\;(l)\to {\mathrm{Ca(OH)}}_{2}\;(s)+\mathrm{HCl}\;(\mathrm{aq})\text{,}$$

and exposing iron power to air,

$$\mathrm{Fe}\;(s)+3O_{2}\;(g)\to {\mathrm{Fe}}_{2}O_{3}\;(s)$$

The latter is used in some types of hand warmer pouches [5]. Huang et al. [29] employed a commercial ‘toe warmer’ composed of a mixture of iron powder, salt, activated carbon, cellulose, and vermiculite that, when exposed to air, maintained a 65 °C ± 2 °C temperature for 55 min to incubate a microfluidic chip hosting an isothermal helicase-dependent amplification (HDA) to detect *C. difficile* in stool samples. The heating reaction was performed in a Styrofoam cup, and the temperature and stability were controlled by the number of holes in the cup. Shaw et al. [30] employed an analogous exothermic Mg reaction with methanol (instead of water):

$$\mathrm{Mg}\;(s)+2\;{\mathrm{CH}}_{3}\mathrm{OH}\;(l)\to \mathrm{Mg}({\mathrm{CH}}_{3}O{)}_{2}\;(s)+H_{2}\;(g)$$

The temperature is buffered by the evaporation of methanol (boiling point 64.7 °C, latent heat of vaporization = 1100 J/g-MeOH) in place of including a solid PCM. Buser et al. [27] compared the ‘energy capacity’ [kJ/g] of various chemical heating reactions, ranked from highest to lowest (with a few additions): Li + H_2_O (32 Jg^−1^), iron filings exposed to air, Fe + 3O_2_ (30 kJ${\cdot g}^{-1}$), Mg:Fe + H_2_O (15 kJ${\cdot g}^{-1}$), CaO + H_2_O (1 kJ${\cdot g}^{-1}$), CaCl (1 kJ${\cdot g}^{-1}$), and Na^+^ + C_2_H_3_O_2_ (0.3 kJ${\cdot g}^{-1}$). For comparison, Buser also includes two batteries: AA alkaline (0.4 kJ g^−1^) and lithium coin cells (0.7 kJ g^−1^).

In addition to the exothermic oxidation reactions listed above, the crystallization of salts from a melted phase can also be used for heating. (This type of heating could also be classified as a phase change, see below.) For example, in some types of hand warmer pouches, sodium acetate trihydrate (SAT) is heated by immersion in hot water. Upon cooling to room temperature, a supercooled liquid persists. A metal piece contained inside the pouch is flexed to spall metal particles that act as nucleation centers, precipitating crystallization and the release of latent heat to maintain the temperature at its melting point. Unfortunately, the melting point of SAT (57 °C) is a few degrees short of that typically used for LAMP, but it can otherwise be used for RPA [25]. A self-contained CRISPR-based SARS-CoV-2 diagnostic system employing incubation by way of a hand warmer has been described by Li et al. [31].

Miniature heaters using lithium metal molded in channels of a laser-machined acrylic piece were developed by Udugama et al. [32]. The acrylic piece was placed in a container with water, and the sample reaction tubes were immersed in a lithium-heated water bath. The temperature was controlled through modulating the lithium–water reaction kinetics by controlling the interface between the reactants (lithium and water) and products (hydrogen bubbles and lithium hydroxide) through a combination of channel dimensions and the addition of excipients. Temperatures between 37 and 65 °C were achieved by tuning the dimensions of the channels. The comparatively high heat of reaction between lithium and water allows for much smaller heaters, with faster heat-up times. Feng et al. [33] used a CaO-fueled chemical heater to incubate an RPA reaction, whereupon amplicons were detected on a lateral flow strip.

For applications requiring low incubation temperatures (<40 °C), such as RPA and related methods, chemical heating without a PCM may be viable. For example, Wang et al. [34] described a diagnostic heater that used a mixture of aluminum powder, CaO, CaCO_3_, CaOH, NaCO_3_, and NaOH wrapped in a fabric and sealed in a polypropylene film packet. To heat a vessel containing reaction tubes, the encapsulating film of the packet was removed and placed in a vessel to which water was added, sustaining the incubation temperature for approximately 30 min. As mentioned above, the latent heat of crystallization can also be used for heating. For example, sodium acetate solutions can be heated by immersion in hot water. During cooling, the temperature is retained (without exothermal chemical reactions), which can also be used to maintain high temperatures after heating. Vloemans et al. [35] demonstrated a compact system where sodium acetate crystallization was used to maintain steady incubation temperatures ranging between 37 and 42 °C.

## 3. Phase-Change Materials (PCMs)

The most common phase-change materials are paraffin wax and other similar organic compounds where the melting temperature is a function of molecular weight. Importantly, the PCM does not exhibit significant supercooling. Some feasible PMCs include Carbowax™ (polyethylene glycol, Dow Chemical), synthetic Polyesterwax (Electron Microscopy Sciences, Hatfield, PA, USA), palmitic acid [24], and various proprietary PCMs surveyed by Singleton et al. [30]. At least several groups have used PureTemp™ PCMs with latent heats of 200 J/g, available in over 200 formulations, with phase change transition temperatures ranging from −40 °C to 150 °C. These PCMs are made from agricultural sources and are biodegradable and nontoxic. One drawback of organic PCMs is their low thermal conductivity, which can be mitigated by adding high-conductivity fillers such as metal particles or carbon particles, but this may reduce the effective latent heat. Additionally, several non-instrumented POC diagnostic systems do not utilize a separate exothermic reaction. Instead, the phase-change material, enclosed in a plastic pouch, is heated in boiling or otherwise hot water, or by an electrical heater, and then contacted with the sample. For example, Lillis et al. [25] incubated RPA reactions in 0.2 µL PCR tubes by placing them in tubes filled with sodium acetate that had been immersed in 70 °C water.

## 4. Estimations

Some illustrative calculations provide estimates of the amount of fuel and PCM needed for chemically heating LAMP reactions. For adiabatic heating (no heat losses), a simple energy balance, assuming that the chemical heating reaction goes to completion, is given by

(1) $$m_{F}{\Delta H}_{rxn}=\Delta T\left[m_{F}C_{F}+m_{W}C_{W}+{{m_{PCM}C_{PCM}+m}_{S}C}_{S}\right]+fm_{PCM}L_{PCM}$$

where $m_{F}$ is the mass of fuel (e.g., Mg powder), $m_{W}$ is the mass of water added, and $m_{S}$ is the mass of the sample(s) and container(s) and any other supporting parts. $C_{F},\;C_{F}\;,and\;C_{S},$ are the respective heat capacities, and ${\Delta H}_{rxn}$ is the heat of reaction (per gram of fuel). $L_{PCM}$ is the latent heat of melting of the PCM, and f is the fraction melted of the PCM. $∆T=\left(T_{A}-T_{0}\right)$ is the increase in temperature from the ambient $T_{0}$ to the amplification temperature $T_{A}$. The above energy balance indicates that the heat of reaction raises the temperature of the components ∆T and melts the PCM, assumes the reaction products have the same heat capacities as the reactants, and neglects gaseous products (e.g., hydrogen or CO_2_) that escape the reactor. The heat capacities for Mg and Mg oxides are about 1 J·g^−1^·C^−1^, 4.2 J·g^−1^·C^−1^ for H_2_O, and 2.5 J·g^−1^·C^−1^ for common phase-change materials. Plastics have heat capacities of about 1.5 J·g^−1^·C^−1^. A stoichiometric amount of water added to Mg (1 mole of Mg: 2 moles of H_2_O) implies 1.45 g (mL) of H_2_O added per 1 g of Mg: $m_{W}=1.45m_{F}.$ To speed up the heating, an excess of water may be used. The ${\Delta H}_{rxn}$ of the reaction is 350 kJ/mole-Mg = 1.4 × 10^4^ J/g-Mg. The latent heat$\;L_{PCM}$ of typical PCMs is 200 J/g. The mass of the sample (mostly water) and container (tube or chip), plus other plastic fixtures, might range from 1 to several grams, depending on the design of the system.

The energy balance indicates the amount of fuel (grams of Mg) needed to heat the system to the target amplification temperature and melt a specified amount of PCM. Ideally, all of the PCM is melted (f=1). More explicitly,

(2) $$m_{F}=\frac{\left(m_{s}C_{s}+m_{w}C_{w}\right)\left(T_{R}-T_{A}\right)+m_{PCM}\left[fL_{PCM}+C_{PCM}\left(T_{A}-T_{0}\right)\right]}{{\Delta H}_{rxn}-C_{F}\left(T_{R}-T_{A}\right)}$$

The amount of PCM needed to be melted is determined by the rate of heat loss and the required reaction incubation time, as the latent heat liberated by the solidification of the PCM must compensate for heat loss in order to maintain a constant amplification temperature.

The rate of the heat loss of the reactor due to thermal conduction ${\dot{Q}}_{ins}$ [W = J·s^−1^] through the insulating layers is

(3) $${\dot{Q}}_{ins}=kA_{ins}\frac{T_{A}-T_{0}}{d}$$

where k [W·cm^−1^ °C^−1^] is the thermal conductivity of the insulator, $A_{ins}$ [cm^2^] is the area of insulation, d [cm] is the thickness of the insulating layer(s), $T_{r}$ [°C] is the amplification temperature (65 °C for LAMP), and $T_{0}$ [°C] is the ambient temperature (20 °C). The rate of convective heat transfer$\;{\dot{Q}}_{con}$ [W] through air-exposed surfaces is

(4) $${\dot{Q}}_{con}=hA_{con}\left(T_{r}-T_{0}\right)$$

where h [W·cm^−2^·C^−1^] is the convective heat transfer coefficient and $A_{con}$ [cm^2^] is the area of air-exposed surfaces. The total rate of heat loss is then

(5) $${\dot{Q}}_{total}={\dot{Q}}_{ins}+{\dot{Q}}_{con}$$

The total energy lost by conduction and convection during incubation time $t_{i}$ [s], e.g., 1 h = 3600 s, is supplied by the latent heat of melting of the PCM.

(6) $$m_{PCM}\cdot L\cdot \;f={\dot{Q}}_{total}\cdot t_{i}=\left({\dot{Q}}_{ins}+{\dot{Q}}_{con}\right)\cdot t_{i}$$

for Styrofoam insulation k=0.03 W·cm^−1^·°C^−1^. The convective heat transfer coefficient *h* is quoted over a wide range, from 0.01 to 1 [W·cm^−2^·°C^−1^], with typical values from 0.25 to 0.5. The parameters ($A_{ins},\;A_{con},\;d$) are dependent on the design.

(7) $$m_{PCM}=\frac{\left[kA_{ins}\frac{T_{r}-T_{0}}{d}+hA_{con}\left(T_{r}-T_{0}\right)\right]\cdot t_{i}}{L_{PCM}f}$$

Assuming areas of $A_{ins}$= 10 cm^2^, $A_{con}$ = 25 cm^2^, d = 1 cm, and $t_{i}=$ 3600 s (1 h), the mass of phase-change material is about 10 g. For 5 mL of water added, the estimated amount of Mg needed (Equation (1)) is about 0.25 g. This is close to values reported by Singleton et al. [22] (6 g PCM, 0.25 g Mg) and Liu et al. [36] (1.5 mL H_2_O, 0.36 Mg, ~0.5 g PCM), where a vacuum Thermos^®^ bottle contained the heater, but lower than values given by Li et al. [37] (10 g PCM, 1 g Mg, and 5 mL H_2_O), where Styrofoam was used as insulation. Discrepancies from estimates might be due to higher heat losses, incomplete reactions, or Mg powder being partially oxidized prior to the test. This also suggests that with better insulation to reduce heat loss, there is some latitude for further miniaturization of the heater apparatus and a reduction in the amount of Mg powder and PCM needed.

## 5. Performance

**Figure 2:**
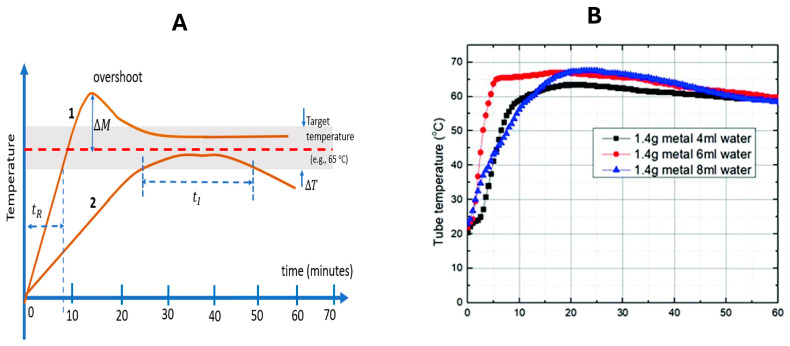
(**A**) Sample time–temperature profiles for chemical self heating showing various figures of merit: rise time $t_{R}$, overshoot ∆M, tolerance band ΔT, and incubation time $t_{I}$ (time spent within tolerance temperature range). Two example cases: Curve 1 shows a fast ramp-up, but with overshoot and a long incubation time. Curve 2 shows a slow ramp-up and insufficient incubation time before cooling below the tolerance band. (**B**) Example of the optimization of the time–temperature in the system shown: 10.5 g of PCM, 1.4 g of Mg:Fe powder, and 4 mL, 6 mL, and 8 mL of water added [37].

The time-temperature profile of chemical heating (Figure 2A), with a target incubation temperature $T_{I}\;$(e.g., 65 °C for LAMP, as shown), suggests some figures of merit. The rise time $t_{r}$ is defined as the time needed to go from $0.1T_{I}$ to $0.9T_{I}$. A rapid increase in temperature shortens the total test time and reduces nonspecific amplification. An excessive overshoot, ∆M, can denature enzymes and needs to be minimized. In the case of LAMP, temperatures in excess of 70 °C can significantly deactivate the polymerases [38]. The tolerance band ΔT is the deviation or variation of the plateau temperature about the target temperature, which should typically be approximately 5 °C or less, as the operating temperature range of LAMP is 60 °C to 65 °C [18,39]. The incubation time $t_{I}\;$is the duration the temperature stays within the tolerance band before cooling. A minimum incubation time of 20 min, and perhaps as long as 60 min, may be needed for some applications. Infrared cameras can provide thermal images of heating, incubation, and cooling, showing temperature variations to identify hot spots and excessive heat leakage due to inadequate insulation. Figure 2B shows the experimental ‘tuning’ of a chemical heating system using Mg-Fe (‘metal’) mixed with powdered PCM for several amounts of water added. Another figure of merit is the robustness of the performance for different ambient temperatures and humidities. Finally, the shelf life of the product should probably be at least 1 year, aided, for example, by adequate packaging to prevent oxidation of the fuel.

## 6. Chemical Heating Formats

**Figure 3:**
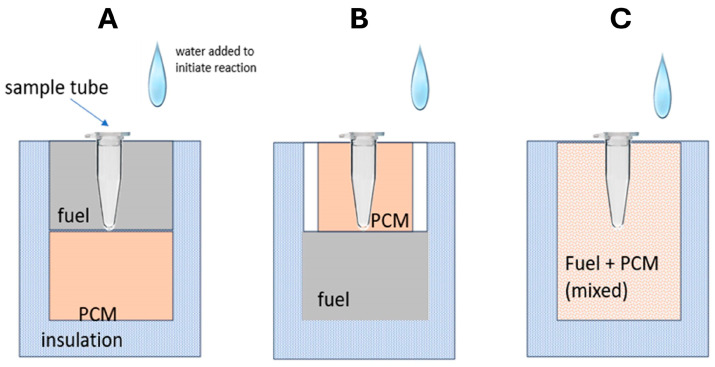
Three configurations for chemical heating with PCMs. (**A**) sample embedded in reactants and temperature buffered by thermal contact with phase-change material (PCM), (**B**) sample surrounded by PCM, which in turn is melted through contact with reactants, and (**C**) mixture of fuel and PCM powders (e.g., Li et al. [37]). Sample can be viewed (to monitor fluorescence of color change) either through tube lid, or from side through viewing port.

Chemical heating methods for ~100 µL reaction tubes, microfluidic chips, and lateral flow strips have been developed. Several configurations for heating a sample are shown in Figure 3. Initially, and most commonly, the reaction and PCM are separated but in close thermal contact. The sample can be surrounded by the reacting mixture or, more commonly, by the PCM. The reaction essentially melts the PCM, and as the reaction progresses, the slowly resolidifying PCM maintains the sample temperature. To moderate and prolong the reaction, water is continually delivered to the fuel, such as by seepage through a porous wick connecting the reaction chamber to a water reservoir.

Liu et al. [36] regulated the exothermic reaction by using a porous material to wick water into the reaction mixture. Good thermal contact is needed between the PCM and the reaction. To improve the thermal conductivity of the PCM, carbon and metal particles have been added to the PCM. Composite paraffin-based PCMs containing expanded graphite and exhibiting improvements with respect to thermal conductivity, homogeneity, and latent heat were described by Ma et al. [40]. The Smart Cup [41] thermos chemical heater was developed into a Smart-*Connected* Cup for POC Zika virus detection [11]. A smartphone seamlessly couples to the smart cup via a holder bracket. A credit-card-sized microfluidic chip (with three parallel LAMP reaction chambers) is incubated by chemical heating. The LAMP reaction includes a bioluminescent reporter (BART, Bioluminescence Assay in Real Time), obviating the need for an excitation source. Luminescence (as a positive test) is detected by a smartphone camera. The smartphone provides signal monitoring and analysis, target quantification, data sharing, and spatiotemporal disease mapping through its GPS.

In an alternate format [37], the Mg/Fe powder is well mixed with PureTemp™ PCM powder (PureTemp LLC, Minneapolis, MN, USA) (with a granule size of 0.4 to 0.6 mm) and packed into a 2.5 cm centrifuge tube with a cutoff length of approximately 5 cm. The tube is placed in a hole formed in a block of Styrofoam. The reaction tubes are inserted into the powder mix with their caps above the surface of the powder mix, and then water is added to initiate the reaction. As water slowly percolates through the powder mix, heat evolves. The formation of hydrogen bubbles also provides a path for water to seep down into the powder mix. The advantage here is simplicity; water is added to the mixture in one dose rather than being dribbled in by wicking, as described above. Moreover, the heat transfer between the reaction and phase-change material is enhanced due to the large interfacial area between the MgFe and PCM granules. The performance was optimized by varying the ratio of MgFe powder to PCM, the total volume of the powder mixture, and the amount of water added. Goertz et al. [42] showed that the ramp-up rate was also sensitive to the NaCl concentration (0 to 1.5%).

Fu et al. [43] described a multistep assay using CaO chemical heating and three PCMs for immunomagnetic separation (37 °C), bacterial lysis and DNA extraction (100 °C), and LAMP (65 °C) in a Thermos^®^-type vacuum bottle, thus demonstrating the chemical heating capability for various sample processing steps. To maintain constant temperatures, chemical-heated systems should be well insulated. Most simply, the system can be packed in Styrofoam, which can be easily cut to shape, leaving access for inserting the sample and ports for visual or optical detector (e.g., cellphone camera) monitoring. Thermos bottles were also used to insulate the reactions. Cup chambers, sample holders, and other structural components can be 3D printed.

More sophisticated applications of chemical heating, including diagnostic assays, utilize phase change partitions. A reaction vessel can be compartmentalized by layers of phase-change materials (various paraffins with distinct melting points) that separate reaction zones. As the temperature increases, the successive partition layers melt, resulting in mixing and the initiation of reactions in stages. Thus, a sequence of reactions and mixing steps can be ‘programmed’ as the heating temperature is slowly increased. Goertz et al. [43] described a multilayered reaction in a PCR tube where the NaCl concentration in the MgFe chemical heating reaction could be controlled and varied to change the temperature ramp from approximately 0.6 °C per minute (0.1% NaCl) to >15 °C per minute (1.5% NaCl); thus, the time between the temperature and activation of PCM melting could be tailored. This work expands the functionality of chemical heating to include initiating mixing and processing, which widens the scope of applications and expands the hands-free automation of diagnostic tests. Table 1 provides a survey of chemical heating methods used for pathogen detection, along with fuel types, reactants, amplification product detection methods, and assay sensitivities.

## 7. Conclusions and Future Directions

This review highlights different strategies and applications of chemical heating in POC medical diagnostic devices that function without electricity. Chemical heating has proven effective for operating molecular diagnostic assays, such as isothermal amplification techniques (like RPA or LAMP) and CRISPR-based tests. Chemical heating offers several benefits over electrical heating for POCT, including the ability to operate without electricity, minimal instrumentation requirements, support for on-site workflows, and low operational costs. Although chemical heating offers several key benefits over electrical heating in POCT, it also has drawbacks. It tends to be relatively slow and can generate hazardous chemical waste, raising concerns about environmental pollution.

Based on the prices for MgFe fuels (as per commercial products sold as Meals Ready-to-Eat) and the amount of PCMs used, the total material cost is about USD 0.10 per test. We also note that many approaches heat a much larger mass of material than the reaction volume, so there is ample room for continued miniaturization, especially with microfluidic formats. The use of phase-change materials as temperature-controlled barriers for partitioning allows multi-stage reactions at several temperatures.

The current challenges in using chemical heating include the miniaturization of chemical heating for palm-sized microfluidic chips or cassettes and the creation of more automated methods of adding water to the reaction, e.g., by depressing pouches integrated in the chip or by other means of providing self-actuated water reservoirs. 

Based on this review, developing a two-stage nested isothermal amplification method that utilizes RPA at 42 °C, followed by LAMP at 60–65 °C to enhance the sensitivity by ~10-fold [45,46,47], enabled by chemical heating, should be feasible and of practical interest. Additionally, integrating a streamlined nucleic acid extraction method with chemical heating-based isothermal amplification could create an efficient molecular screening test.

## Figures and Tables

**Table 1:** Chemical heating for POC molecular detection of pathogens.

Isothermal Amplification Assay	Pathogen (s)	Fuel	Reactants	Time to Chemical Heating	Amplification Time	Amplified Product Detection	Analytical Sensitivity	The Used Device	Reference
1—LAMP	Zika virus	Magnesium Iron	Mg-Fe + H_2_O	10 min	60 min	Bioluminescence	5 PFU/sample	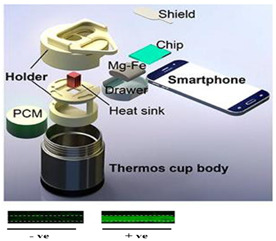	[11]
2—LAMP	Zika virus	Magnesium Iron	Mg-Fe + H_2_O	10 min	40 min	Colorimetric detection—intercalating dye Leco Crystal Violet (LCV)	5 PFU/sample	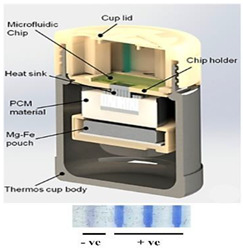	[12]
3—LAMP	*Plasmodium falciparum* malaria	Calcium oxide	CaO + H_2_O	15 min	45 min	Turbid visual detection and fluorescent (Calcein) detection	50 copies/µL	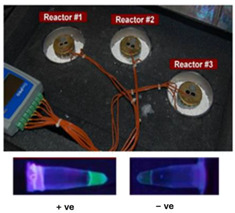	[20]
4—LAMP	*Plasmodium falciparum* malaria	Calcium oxide	CaO + H_2_O	15 min	60 min	Turbid visual detection and fluorescent (Calcein) detection	50 copies/µL	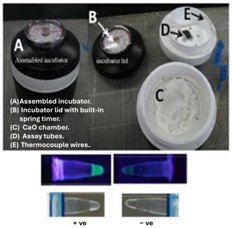	[21]
5—LAMP	HIV-1	Magnesium Iron	Mg-Fe + H_2_O	12 min	60 min	Nucleic acid lateral flow detection	75 copies/reaction	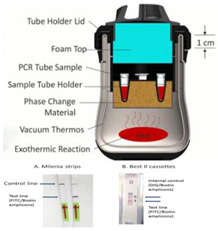	[23]
6—LAMP	HIV-1	Magnesium Iron	Mg-Fe + H_2_O	11 min	60 min	BHQ1-labeled quencher probe	3115 copies/mL	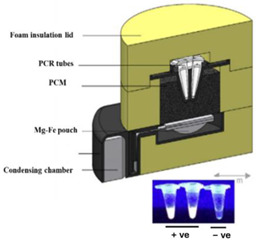	[24]
7—RPA	HIV-1	Sodium acetate	Na^+^ + C_2_H_3_O_2_	1 min	20–30 min	Nucleic acid lateral flow detection	10 copies/µL	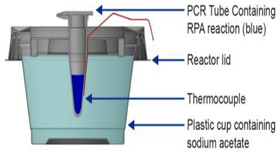	[25]
8—LAMP	Filarial parasites (*B*. *malayi*, *O*. *volvulus*, and *W*. *bancrofti)*	Magnesium Iron	Mg-Fe + H_2_O	15 min	40–70 min	Colorimetric detection—proton detection (pH-sensitive indicator dyes)	*B*. *malayi* 1.0 pg DNA/reaction, *O*. *volvulus* 0.01 ng DNA/reaction, and W. bancrofti 0.1 pg DNA/reaction	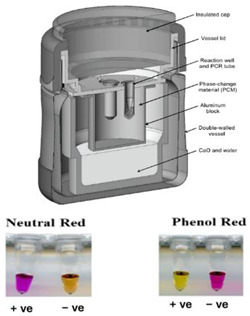	[26]
9—HDA	*Clostridium difficile* (*C. difficile*)	Iron filings	Fe + 3 O_2_	22 min	30 min	Gel electrophoresis analysis	1.25 × 10^−2^ pg/reaction	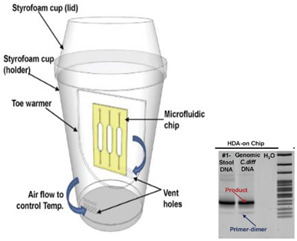	[29]
10—RPA/CRISPR	SARS-CoV-2	Iron filings	Fe + 3 O_2_	2 min	15 min	Nucleic acid lateral flow detection	100 copies/reaction	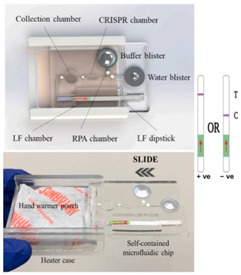	[31]
11—RPA	T4 bacteriophage	Lithium	Li + H_2_O	1 min	15 min	- Fluorescent test (Taqman probe) - Gel electrophoresis	NA	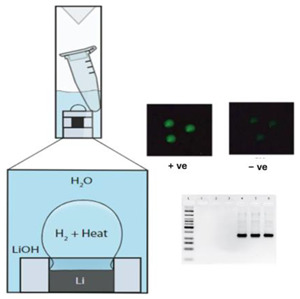	[32]
12—RPA	Enterohemorrhagic *Escherichia coli* O157:H7	Calcium oxide	CaO + H_2_O	9 min	15 min	Nucleic acid lateral flow detection	36.23 CFU/mL	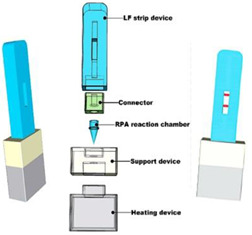	[33]
13—LAMP	*Cronobacter species*	Calcium oxide	CaO + H_2_O	7 min	60 min	HNB (Hydroxy naphthol blue)—Metal indicator	4.2 × 10^2^ cfu/g	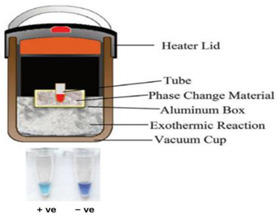	[43]
14—LAMP	Herpes simplex virus type 2 (HSV-2)	Magnesium Iron	Mg-Fe + H_2_O	10 min	60 min	Fluorescent intercalating dyes	4.2 PFU/sample	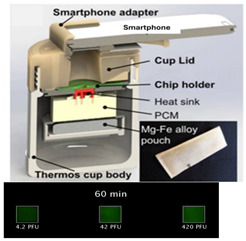	[41]
15—LAMP	SARS-CoV-2	Magnesium Iron	Mg-Fe + H_2_O	3 min	30 min	- pH-sensitive indicator dyes (Phenol red) - Fluorescent intercalating dyes	10 copies/reaction	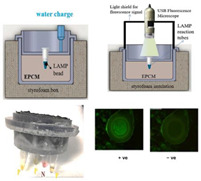	[37]
16—LAMP	HIV-1	Calcium oxide	CaO + H_2_O	10–15 min	60 min	Fluorescence detection using labeled primer along with a quencher probe	HIV- 1 DNA: 10 copies/reaction HIV-1 RNA: 140 copies/reaction	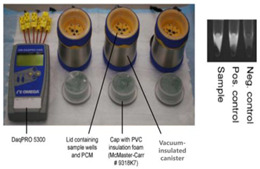	[44]

NA = Not applicable.

## Data Availability

Data are contained within the article.
